# Structural studies of a glycoside hydrolase family 3 β-glucosidase from the model fungus *Neurospora crassa*

**DOI:** 10.1107/S2053230X18015662

**Published:** 2018-11-26

**Authors:** Saeid Karkehabadi, Henrik Hansson, Nils Egil Mikkelsen, Steve Kim, Thijs Kaper, Mats Sandgren, Mikael Gudmundsson

**Affiliations:** a Department of Molecular Sciences, Swedish University of Agricultural Sciences, PO Box 7015, SE-750 07 Uppsala, Sweden; b DuPont Industrial Biosciences, 925 Page Mill Road, Palo Alto, CA 94304, USA

**Keywords:** glycoside hydrolase family 3, β-glucosidase, biodegradation, crystal structure, *Neurospora crassa*, *Nc*Cel3A

## Abstract

The glycoside hydrolase family 3 (GH3) β-glucosidases are a structurally diverse family of enzymes. Cel3A from *Neurospora crassa* (*Nc*Cel3A) belongs to a subfamily of key enzymes that are crucial for industrial biomass degradation. β-Glucosidases hydrolyse the β-1,4 bond at the nonreducing end of cellodextrins. The hydrolysis of cellobiose is of special importance as its accumulation inhibits other cellulases acting on crystalline cellulose. Here, the crystal structure of the biologically relevant dimeric form of *Nc*Cel3A is reported. The structure has been refined to 2.25 Å resolution, with an *R*_cryst_ and *R*_free_ of 0.18 and 0.22, respectively. *Nc*Cel3A is an extensively N-glycosylated glycoprotein that shares 46% sequence identity with *Hypocrea jecorina* Cel3A, the structure of which has recently been published, and 61% sequence identity with the thermophilic β-glucosidase from *Rasamsonia emersonii*. *Nc*Cel3A is a three-domain protein with a number of extended loops that deepen the active-site cleft of the enzyme. These structures characterize this subfamily of GH3 β-glucosidases and account for the high cellobiose specificity of this subfamily.

## Abstract 2

The glycoside hydrolase family 3 β-glucosidase Cel3A from *Neurospora crassa* (*Nc*Cel3A) is highly specific for cellobiose. The crystal structure of *Nc*Cel3A has been solved to 2.25 Å resolution. The structure is a dimer and exhibits a high degree of N-glycosylation.

## 1. Introduction   

Fungal degradation of cellulose is considered to be accomplished by four primary enzymatic activities, which act synergistically to overcome the recalcitrance of the cellulose polymer (Payne *et al.*, 2015 ▸). Endoglucanases [EGs; endo-(1,4)-β-d-glucanhydrolases; EC 3.2.1.4] cleave exposed cellulose chains randomly, which introduces new chain ends, and release shorter cello-oligosaccharides of varying lengths. Cellobiohydrolases [CBHs; exo-(1,4)-β-d-glucan cellobio­hydrolases; EC 3.2.1.91 and EC 3.2.1.176] processively traverse a cellulose chain while successively releasing cellobiose units. β-Glucosidases (BGLs; EC 3.2.1.21) hydrolyse the soluble oligosaccharides and cellobiose to primarily produce glucose. Lytic polysaccharide monooxygenases (LPMOs) are a more recently discovered nonhydrolytic class of polysaccharide-degrading enzymes (Harris *et al.*, 2010 ▸). LPMOs break polysaccharide chains in an oxygen- and electron-dependent process; they break glycosidic bonds by directly oxidizing the C1 or C4 carbon of a glycopyranose ring, apparently without the need for depolymerization (Meier *et al.*, 2018 ▸). In the classification system of carbohydrate-active enzymes, the Carbohydrate-Active enZYmes Database (CAZy; Lombard *et al.*, 2014 ▸), β-glucosidases can be found in glycoside hydrolase (GH; Henrissat & Davies, 1997 ▸) families GH1, GH3, GH5, GH9, GH30 and GH116. The enzymes in all of these GH families except for GH9 perform hydrolysis by a double-displacement reaction mechanism with net retention of the configuration at the anomeric carbon (Gebler *et al.*, 1992 ▸). All such β-glucosidases have a common (α/β)_8_ TIM-barrel fold. Glycoside hydrolase family 3 (GH3) is one of the larger families in the CAZy classification and currently contains over 13 600 annotated protein sequences. The family groups together several exo-acting activities and includes enzyme members with broad substrate specificity with respect to the type of monosaccharide, linkages and chain length of the substrate.

The filamentous fungus *Neurospora crassa* is an ascomycete that decomposes and consumes dead plant material in nature. It has been widely used as a model organism in the field of eukaryotic biology (Davis & Perkins, 2002 ▸) and produces and secretes a full suite of carbohydrate-degrading enzymes (Romero *et al.*, 1999 ▸; Eberhart *et al.*, 1964 ▸, 1977 ▸; Yazdi *et al.*, 1990 ▸). These enzymes are able to completely decrystallize and depolymerize cellulose as well as other plant cell-wall polysaccharides in an orchestrated fashion (Tian *et al.*, 2009 ▸). There are at least seven genes encoding GH3 enzymes in the genome of *N. crassa*. Three of these genes have a signal peptide and are expected to produce secreted GH3 enzymes: GH3-1 (Bgl7, NCU03641), Cel3A (GH3-3, Bgl6, NCU08755) and GH3-4 (Bgl2, NCU04952). All three gene products are upregulated when wild-type *N. crassa* is grown using cellulose as the main carbon source (Wu *et al.*, 2013 ▸), but only Cel3A and GH3-4 have been experimentally characterized as true β-glucosidases (Tian *et al.*, 2009 ▸; Bohlin *et al.*, 2010 ▸), and it has recently been shown that Cel3A exhibits a high affinity for cellobiose compared with longer β-1,4-gluco-oligosaccharides (Colabardini *et al.*, 2016 ▸). Cel3A was identified in the conidia cell walls of *N. crassa* (Maddi *et al.*, 2009 ▸) and GH3-4 was identified in the supernatant of *N. crassa* grown on Avicel and *Miscanthus* by mass spectrometry (Tian *et al.*, 2009 ▸). Cel3A is homologous to several enzymes with recently published crystal structures: *Rasamsonia emersonii* Cel3A (*Re*Cel3A; Gudmundsson *et al.*, 2016 ▸), *Aspergillus aculeatus* BGLI (*Aa*BGLI; Suzuki *et al.*, 2013 ▸), *Aspergillus oryzae* Cel3A (*Ao*Cel3A; Agirre *et al.*, 2016 ▸) and *Aspergillus fumigatus* Cel3A (*Af*Cel3A; Agirre *et al.*, 2016 ▸). Both *Re*Cel3A and *Aa*BGLI have also been shown to exhibit the properties of dedicated cellobiases.

There are currently nine structural models of fungal GH3 enzymes available in the Protein Data Bank. Six are characterized as β-1,4-glucosidases and the remaining three are a β-1,3-1,4-glucanase (Varghese *et al.*, 1999 ▸), a β-*N*-acetyl­glucosaminidase (Qin *et al.*, 2015 ▸) and a β-1,4-xylosidase (PDB entry 5a7m; Mikkelsen *et al.*, unpublished work). These structures highlight the modularity of the GH3 enzymes, which has become apparent since the first structure of a GH3 enzyme was solved, that of the exo-β-1,3-1,4-glucanase *Hordeum vulgare* ExoI (*Hv*ExoI). *Hv*ExoI is composed of two domains: an N-terminal (α/β)_8_ TIM-barrel domain containing the catalytic nucleophile aspartate and an (α/β)_6_ sandwich domain containing the catalytic acid/base glutamate. This two-domain structure and the position of the catalytic residues are a core feature of GH3 enzymes and are retained in the multidomain GH3 enzymes that are now known. In 2010 the first three-domain GH3 structure was published, that of the β-glucosidase *Thermotoga neapolitana* Bgl3B (*Tn*Bgl3B; Pozzo *et al.*, 2010 ▸). The C-terminal FnIII-like third domain straddles the barrel and sandwich domains on the opposite side to the active site of the enzyme. The function of the third domain is unknown, although it has been suggested that it stabilizes the TIM-barrel domain, which has an incomplete/collapsed fold in all three-domain GH3 enzymes with known structure (Gudmundsson *et al.*, 2016 ▸). A fourth domain is present in *Kluyveromyces marxianus* BglI (*Km*BglI; Yoshida *et al.*, 2010 ▸) and *Pseudoalteromona* sp. ExoP (*Ps*ExoP; Nakatani *et al.*, 2012 ▸). *Km*BglI has a PA14 domain that extends the active site, while *Ps*ExoP has a highly mobile CBM-like domain of unknown function.

In this study, we present the crystallization and structure determination of a GH3 β-glucosidase from *N. crassa* (*Nc*Cel3A) solved to 2.25 Å resolution. These results are discussed in the light of differences from and similarities to other GH3 enzymes with known structure. *Nc*Cel3A is most similar to the recently solved crystal structures of the GH3 β-glucosidases *Aa*BGL1 and *Re*Cel3A (PDB entries 4iib and 5ju6; Suzuki *et al.*, 2013 ▸; Gudmundsson *et al.*, 2016 ▸). These three GH3 structures all have certain major structural features in common. A high number of N-glycosylations can be observed, with over 40 modelled glycans per protein molecule, which are peculiarly localized only on one face of the proteins. These enzymes also all have an extended C-terminal loop protruding from the C-terminal domain covering large parts of the first domain and several of its N-glycosylations. The linkers connecting the three domains of these enzymes extend much further towards the active-site cleft of the enzyme compared with *Hj*Cel3A and *Tn*Bgl3B, and unlike *Hj*Cel3A and *Tn*Bgl3B this class of GH3 β-glucosidases have all been shown to exist as dimers in solution (Murray *et al.*, 2004 ▸; Gudmundsson *et al.*, 2016 ▸; Suzuki *et al.*, 2013 ▸; Agirre *et al.*, 2016 ▸).

## 2. Materials and methods   

### 2.1. Macromolecule production   

The *gh3-3* gene encoding *Nc*Cel3A (GenBank EAA26868.1) was overexpressed in an *H. jecorina* strain with eight genes coding for cellulases (*cbh1*, *cbh2*, *egl1*, *egl2*, *egl3*, *egl4*, *egl5* and *egl6*) deleted and one gene coding for a mannanase (*man1*) deleted. The target gene was cloned into the pTrex3G vector (amdS^R^, amp^R^, P_cbh1_; Foreman *et al.*, 2005 ▸) and used to transform *H. jecorina*. Transformants of *H. jecorina* were picked from Vogel’s minimal medium plates (Vogel, 1956 ▸) containing acetamide after seven days of incubation at 37°C and were grown in Vogel’s minimal medium with a mixture of glucose and sophorose as carbon sources. The overexpressed protein appeared as a dominant protein in the culture supernatants.

Culture filtrate from the production of *Nc*Cel3A in *H. jecorina* (obtained using Sarstedt Filtropur 0.2 µm filters) was diluted tenfold with 25 m*M* sodium acetate pH 4.0 and incubated at 37°C for 30 min. The sample was desalted using a Sephadex G-25M column (GE Healthcare, Piscataway, New Jersey, USA) equilibrated with acetate buffer and concentrated using a centrifugal concentrator with a 10 kDa cutoff (Vivascience, Littleton, Massachusetts, USA). The protein solution was loaded onto a gel-filtration column (Superdex 200 HiLoad 16/60) with 20 m*M* sodium acetate pH 7.5, 150 m*M* NaCl as the running buffer and eluted at a flow rate of 0.5 ml min^−1^ on an ÄKTApurifier (GE Healthcare Bio­sciences, Sweden) at room temperature. The fractions containing *Nc*Cel3A were pooled, washed and concentrated to 15 mg ml^−1^ in 20 m*M* sodium acetate buffer pH 5.0, 20 m*M* NaCl using Vivaspin 20 centrifuge concentration tubes with a 30 kDa molecular-mass cutoff (Sartorius Stedim Biotech, France). The purity of the *Nc*GH3 protein was greater than 95% as judged by SDS–PAGE. The protein concentration was determined by measuring the absorbance of the protein solution at 280 nm using a calculated extinction coefficient for *Nc*Cel3A of 160 130 *M* ^−1^ cm^−1^.

### 2.2. Crystallization   

Crystals of *Nc*Cel3A were grown using the hanging-drop vapour-diffusion method at 20°C. Initial crystallization trials were carried out using a Mosquito crystallization robot (TTP Labtech, Cambridge, England). To identify the best crystallization condition, several commercially available crystallization screens such as PEG/Ion (Hampton Research), Crystal Screen and Crystal Screen 2 (Molecular Dimensions, UK) and the JCSG+ Suite (Qiagen, Germany) were utilized. Using a 96-well plate, drops (0.3 µl) were prepared by mixing protein solution at ∼15 mg ml^−1^ with an equal amount of well solution. The best crystallization condition was obtained from the PEG/Ion screen and consisted of 0.2 *M* ammonium citrate dibasic pH 5.1, 20%(*w*/*v*) polyethylene glycol 3350. Crystals that were large enough for X-ray data collection grew within a week. Prior to X-ray data collection, crystals of *Nc*Cel3A were transferred into a cryoprotectant solution containing 40% PEG 3350 and cooled in liquid nitrogen.

### 2.3. Data collection and structure refinement   

Data were collected at a wavelength of 1.0 Å at 100 K on beamline I911-3 at MAX-lab, Lund, Sweden. The data were processed using *XDS* (version of 3 February 2010; Kabsch, 2010 ▸) and scaled by the scaling program *SCALA* v.3.3.16 (Evans, 2006 ▸) via the *CCP*4 v.6.5.0 program suite (Winn *et al.*, 2011 ▸). A set of 5% of the reflections was put aside and used to calculate the quality factor *R*_free_ (Brünger, 1992 ▸). Details of data collection and processing are presented in Table 1 ▸. The crystal structure of *Nc*Cel3A was determined by molecular replacement using *Phaser* v.2.1.4 (McCoy *et al.*, 2007 ▸). The molecular-replacement search molecule consisted of one molecule of β-glucosidase 1 from *A. aculeatus* (*Aa*BGL1; PDB entry 4iib; Suzuki *et al.*, 2013 ▸). Rigid-body refinement of individual molecules using data between 20 and 3 Å resolution was performed and the resulting 2*F*_o_ − *F*_c_ and *F*_o_ − *F*_c_ electron-density maps showed continuous density for two *Nc*Cel3A molecules in the asymmetric unit. Throughout the refinement, 2*mF*_o_ − *DF*_c_ and *mF*_o_ − *DF*_c_ σ_A_-weighted maps (Pannu & Read, 1996 ▸) were inspected and the models were manually adjusted during repetitive cycles of iterative model building using *Coot* v.0.8.7 (Emsley *et al.*, 2010 ▸; Emsley & Cowtan, 2004 ▸; Krissinel & Henrick, 2007 ▸) and maximum-likelihood refinement and TLS refinement using *REFMAC*5 v.5.8.0135 (Murshudov *et al.*, 1997 ▸, 2011 ▸) until no further improvement of structural parameters could be observed. Water molecules were added using *ARP*/*wARP* v.7.1 (Perrakis *et al.*, 1997 ▸) and manually using *Coot*. Figures were prepared using *PyMOL* v.1.5.0.4 (DeLano, 2002 ▸). Root-mean-square deviation values (r.m.s.d.s) were calculated using the *SSM* function (Krissinel & Henrick, 2004 ▸) in *Coot*. Carbohydrates were modelled via cyclical building in *Coot* and refinement with *REFMAC*5 with torsion-angle restraints enabled. Validation of correct stereochemistry and low-energy conformation of carbohydrates between refinement cycles was performed with *Privateer* v.MKIII (Agirre *et al.*, 2015 ▸). Coordinates and structure factors have been deposited in the Protein Data Bank (PDB) with PDB code 5nbs.

## 3. Results and discussion   

### 3.1. Expression, purification and crystallization of *Nc*Cel3A   

The purified and concentrated *Nc*Cel3A crystallized in the orthorhombic space group *P*2_1_2_1_2, with unit-cell parameters *a* = 142.9, *b* = 286.8, *c* = 58.1 Å. The molecular-replacement solution gave a best solution with two protein molecules (molecular weight 93.6 kDa) in the asymmetric unit, with a calculated *V*_M_ of 3.17 Å^3^ Da^−1^ (Matthews, 1968 ▸) and a solvent content of 61%. The *Nc*Cel3A structure was refined at 2.25 Å resolution to final *R*_cryst_ and *R*_free_ values of 17.9 and 21.6%, respectively. The final *Nc*Cel3A structure is composed of two noncrystallographic symmetry (NCS)-related molecules with 842 and 843 amino-acid residues, respectively, 875 water molecules and 85 carbohydrate residues. No gaps were found in the protein chains. There are eight *cis*-peptides and eight cysteines, of which six form disulfide bonds, in each protein molecule in the structure. Chains *A* and *B* have 45 and 38 modelled N-glycans (*N*-acetyl-β-d-glucosamine, α-d-mannopyranose and β-d-mannopyranose), respectively. Additional X-ray data-collection and refinement statistics are presented in Table 1 ▸.

### 3.2. The fold and structure of *Nc*Cel3A   

The *Nc*Cel3A crystal structure model is composed of two NCS-related protein molecules in the asymmetric unit. *Nc*Cel3A chain *A* contains 843 amino-acid residues and the first modelled residue is Ser34 of the deposited *Nc*Cel3A DNA sequence (GenBank EAA26868.1), while chain *B* contains 842 residues and the first modelled residue is Leu35. The last modelled residue in both protein chains is Pro875. The signal-peptide cleavage site of the EAA26868.1 sequence predicted by *SignalP* 4.1 (Petersen *et al.*, 2011 ▸) is at position 18. The N-terminal residues 1–16 are most likely not visible owing to high flexibility. The overall structure of *Nc*Cel3A is composed of three separate domains connected by two linkers and has a high degree of N-glycosylation. The two *Nc*Cel3A protein chains form a dimer similar to those observed in *Re*Cel3A (Gudmundsson *et al.*, 2016 ▸) and *Aa*BGL1 (Suzuki *et al.*, 2013 ▸).

There are 15 N*X*T/S N-glycosylation sites in *Nc*Cel3A and all are found on or near the surface of the protein molecule. 12 sites in chain *A* and ten sites in chain *B* possessed sufficient electron density to allow the modelling of N-glycan moieties. The glycans ranged in length from single *N*-acetylglucosamine (GlcNAc) residues to longer Man_7_GlcNAc_2_ chains. The majority of the long N-glycan chains are positioned on domain 1 and domain 2, and are also asymmetrically distributed onto the same face of the protein. This pattern of N-glycans seems to be conserved among this subclass of GH3 BGLs. We have previously theorized (Gudmundsson *et al.*, 2016 ▸) that these extensive and partially buried N-glycans serve a function in stabilizing the collapsed TIM-barrel fold of domain 1 by binding to hydrophobic patches in conjunction with the C-terminal loop V. Also of interest is that protein glycans have a potential binding affinity for polysaccharides such as cellulose, as has been proposed by Payne *et al.* (2013 ▸). This could suggest a functionality of the N-glycans in conferring binding to cellulose, which would be in the process of being degraded by other cellulases and thus be where β-glucosidic enzyme activity would be most needed by the organism. For additional analysis of GH3-BGL N-glycosylation, see Gudmundsson *et al.* (2016 ▸) and Agirre *et al.* (2016 ▸).

The structure of *Nc*Cel3A is highly homologous to several GH-family 3 β-glucosidase structures, *Re*Cel3A, *Aa*BGL1, *Af*Cel3A and *Ao*Cel3A, with 61% sequence identity to the first three proteins and 59% to *Ao*Cel3A. Structural alignments highlight the same high similarities, with r.m.s.d. values of 0.74 Å with regard to *Re*Cel3A and *Af*Cel3A, 0.76 Å for *Ao*Cel3A and 0.82 Å for *Aa*BGL1. All three-domain GH3 enzymes fall into subcluster C2 as specified by Cournoyer & Faure (2003 ▸).

### 3.3. Domain 1   

**Figure 1:**
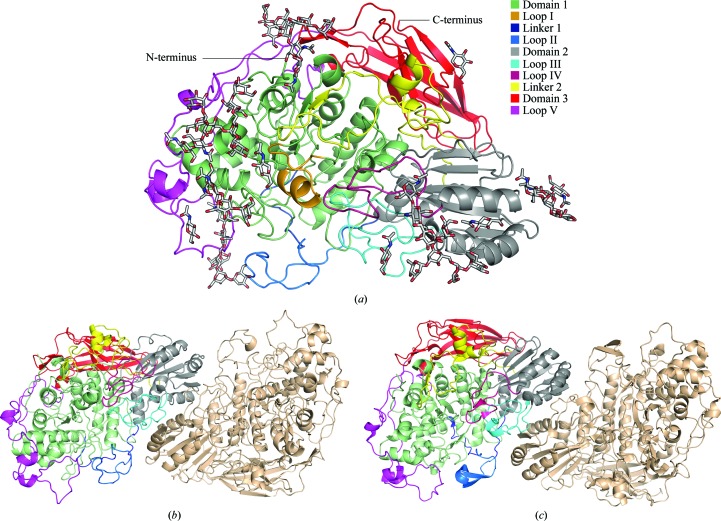
(*a*) Cartoon representation of the *Nc*Cel3A structure displayed in ribbon representation. The three domains of the protein are coloured green (domain 1), grey (domain 2) and red (domain 3). Loops and linkers are highlighted in colours according to the legend in the top-right corner. N-Glycosylations are shown as grey sticks. (*b*, *c*) The quaternary structures of *Nc*Cel3A (*b*) and *Re*Cel3A (Gudmundsson *et al.*, 2016 ▸) (*c*) showing the dimer formation found in these two structures.

**Figure 2:**
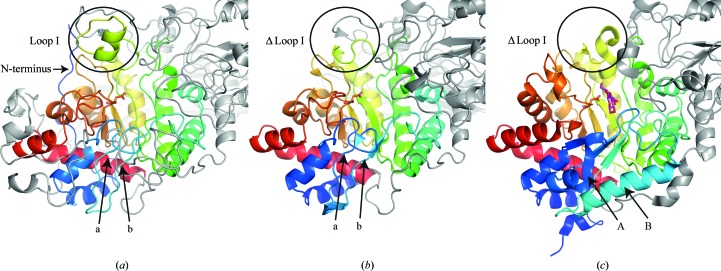
Cartoon representation of domain 1 of (*a*) *N. crassa* Cel3A (*Nc*Cel3A), (*b*) *H. jecorina* Cel3A (*Hj*Cel3A; PDB entry 3zyz; Karkehabadi *et al.*, 2014 ▸) and (*c*) *H. vulgare* ExoI (PDB entry 1iex; Hrmova *et al.*, 2001 ▸) shown in ribbon representation. Loops a and b in *Nc*Cel3A and *Hj*Cel3A highlight the deleted helices which are present and marked A and B in *Hv*ExoI. The position of loop I is highlighted by a circle.

The first domain of *Nc*Cel3A (residues 34–340; coloured light green in Fig. 1 ▸) is composed of a collapsed TIM-barrel fold [or ββ(β/α)_6_ fold], which was described for the first time in the structure of the GH family 3 β-glucosidase *Tn*Bgl3B (Pozzo *et al.*, 2010 ▸) and more recently by other groups presenting new structures of GH3 β-glucosidases (Yoshida *et al.*, 2010 ▸; Suzuki *et al.*, 2013 ▸; Karkehabadi *et al.*, 2014 ▸; Gudmundsson *et al.*, 2016 ▸; Agirre *et al.*, 2016 ▸). Domain 1 of *Nc*Cel3A contains the catalytic nucleophile Asp276, as well as the majority of residues comprising substrate-binding subsite −1, with the catalytic centre being located between subsites −1 and +1. The subsite nomenclature of glycoside hydrolases is described by Biely *et al.* (1981 ▸) and Davies *et al.* (1997 ▸). All three-domain GH3 structures, where the C-terminal domain is an FNIII-like domain, lack two α-helices compared with the canonical TIM barrel of the barley GH3 structure *Hv*ExoI (Varghese *et al.*, 1999 ▸; Fig. 2 ▸). The loss of overall protein stability owing to the lack of central secondary elements may be mitigated by the introduction of a disulfide bridge between the two strands (Cys69 and Cys85 in *Nc*Cel3A). The missing loops allow a wider binding cleft in *Hj*Cel3A, whereas in *Nc*Cel3A this space is occupied, and extended, primarily by loops I and II (Fig. 1 ▸ *a*). The structure of *Nc*Cel3A, as well as those of *Re*Cel3A and *Aa*BGL1, has a protruding loop (loop I in Fig. 1 ▸) which is not present in the three-domain GH3 structures *Hj*Cel3A (Fig. 1 ▸ *b*), *Km*BGLI and *Tn*Bgl3B. This loop extends one side of the active-site cleft, whereas loop II in linker 1 (Fig. 2 ▸) extends the opposite side of the cleft. The α-helical part of loop I introduces three residues towards the active-site cleft: Glu200, Asp203 and Tyr204. In the *Re*Cel3A structure there is a two-amino-acid deletion compared with *Nc*Cel3A and *Aa*Bgl1. The lack of these two amino-acid residues results in a loss of α-helical structure that leaves a slightly wider active-site cleft.

### 3.4. Linker 1 and loop II   

**Figure 3:**
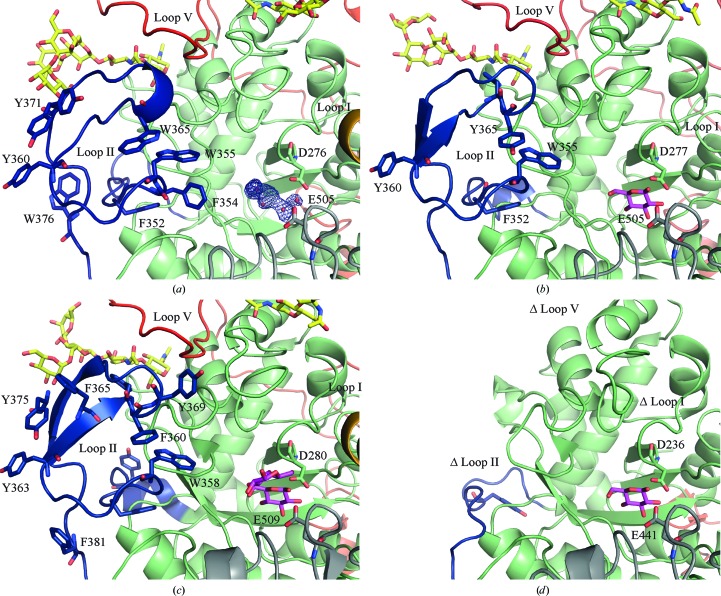
Cartoon ribbon representation of loop II (blue). (*a*) shows *Nc*Cel3A, (*b*) *Re*Cel3A (PDB entry 5ju6; Gudmundsson *et al.*, 2016 ▸), (*c*) *Aa*Bgl1 (PDB entry 4iih; Suzuki *et al.*, 2013 ▸) and (*d*) *Hj*Cel3A (PDB entry 3zyz; Karkehabadi *et al.*, 2014 ▸). Domain 1 is coloured green, domain 2 grey, loop V red and loop I orange; active-site ligands are shown as magenta sticks, active-site residues in stick representation, electron density as a blue mesh and N-glycosylations as yellow sticks.

In *Nc*Cel3A, domain 1 is connected to domain 2 by a 42-residue linker (residues 341–383). In *Hj*Cel3A and *Hv*ExoI this linker is only 18 and 16 residues in length, respectively. The insertion of 25 residues (residues 351–376) observed in *Nc*Cel3A, as well as in *Re*Cel3A and *Aa*Bgl1, and denoted as loop II in Fig. 3 ▸, has previously been described as a hydrophobic linker that activates *T. aurantiacus* BGLII in organic solvents (Hong *et al.*, 2006 ▸) and is present with a similar fold in the *Re*Cel3A and *Aa*Bgl1 structures (Yoshida *et al.*, 2010 ▸; Suzuki *et al.*, 2013 ▸; Karkehabadi *et al.*, 2014 ▸; Gudmundsson *et al.*, 2016 ▸). Loop II is not present in the other fungal GH3 structures *Hj*Cel3A (Fig. 3 ▸ *d*) and *Km*BGLI, but in the *Nc*Cel3A structure it extends the opposite side of the active-site cleft compared with loop I. In *Nc*Cel3A, loop II has two tryptophan residues and one phenylalanine (Trp355, Trp365 and Phe354), which constitute one side of the putative substrate-binding subsites +1 and +2 (discussed in more detail in Sections 3.7 and 3.8). Loop II also contains two tyrosine residues and a tryptophan (Tyr360, Tyr371 and Trp376), which are positioned outside the active-site cleft. Interestingly, this part of loop II resembles the flat binding surface of a carbohydrate-binding molecule type 1 (CBM1), which also consists of two tyrosines and a tryptophan (Mattinen *et al.*, 1998 ▸). There seems to be high variability in loop II among GH3 β-glucosidases. The two aromatic residues Phe352 and Trp355 are the most conserved residues in this loop, and these two residues are also present in the *R. emersonii* and *A. aculeatus* structures (Figs. 3 ▸ *a*, 3 ▸ *b* and 3 ▸ *c*). All of the GH3 structures that include loop II have certain common features. For instance, Tyr360 and an aromatic residue at the position of Trp366 are present in these structures. A second common feature is the presence of an N-glycan chain emanating from Asn57 in *Nc*Cel3A, which is wedged in between loop II and the C-terminal loop V. This apparent feature of using N-glycans as a structural element could be unique to this class of enzymes.

### 3.5. Loops in domain 2 and the second linker   

**Figure 4:**
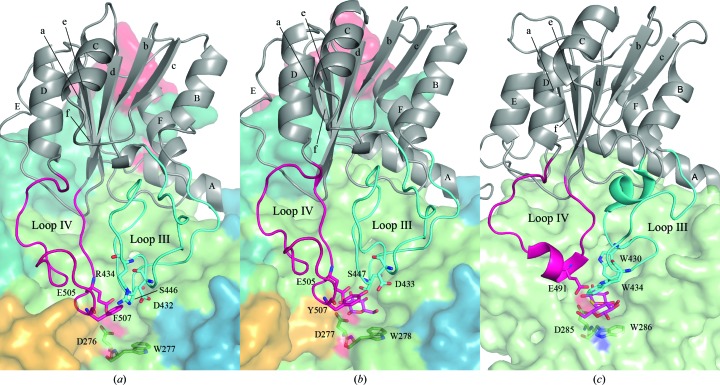
Overview of domain 2 (grey) in ribbon representation. (*a*) shows *Nc*Cel3A, (*b*) *Re*Cel3A (PDB entry 5ju6; Gudmundsson *et al.*, 2016 ▸) and (*c*) *Hv*ExoI (PDB entry 1iex; Hrmova *et al.*, 2001 ▸). Loops III and IV are highlighted in cyan and magenta, respectively. Other domains are represented by surfaces coloured according to the scheme presented in Fig. 1 ▸.

The second domain of the *Nc*Cel3A structure (residues 383–584) has an (α/β)_6_ sandwich fold, which is structurally well preserved among all GH3 enzymes with known structure that contain at least two domains, except in the bacterial *Tn*Bgl3B, in which one edge β-strand (strand c in Fig. 4 ▸) is substituted by an additional α-helix and a flexible loop (Pozzo *et al.*, 2010 ▸). Domain 2 of *Nc*Cel3A has two loops, III and IV, that encompasses residues 421–455 and 501–524, respectively. These two loops constitute one side of the active-site cleft (Fig. 4 ▸), in between loops I and II. Loop III of *Nc*Cel3A is a 34-residue loop that extends between strand b and helix B. The loop folds back over itself and is stabilized by a disulfide bridge formed by Cys430 and Cys435. Ser446 and Asp432 are two conserved residues in loop III that are directed towards the active site. Ser446 is especially important as it is positioned pointing directly towards the hexose ring of a substrate bound in the −1 subsite. In *Hv*ExoI this position is occupied by Trp343 (Fig. 4 ▸ *c*), which is a very common motif in carbo­hydrate-binding domains. Trp343 constitutes half of the ‘coin-slot’ binding pocket of *Hv*ExoI (Varghese *et al.*, 1999 ▸), which is not present in any three-domain GH3 structures. Loop IV is a 23-residue loop in which the catalytic acid/base Glu505 is located. *Nc*Cel3A has a phenylalanine at position 507, whereas the other related GH3 structures have a tyrosine located at this position.

Domain 2 of the *Nc*Cel3A structure is followed by the second linker region (residues 585–649). This linker is extended compared with that described as a C-terminal extension in the *Hv*ExoI structure. This extension covers and stabilizes loops I and IV. These loops appear to be a large part of the interface between domains 2 and 3, but are not present in the barley enzyme *Hv*ExoI.

### 3.6. Domain 3 and loop V   

**Figure 5:**
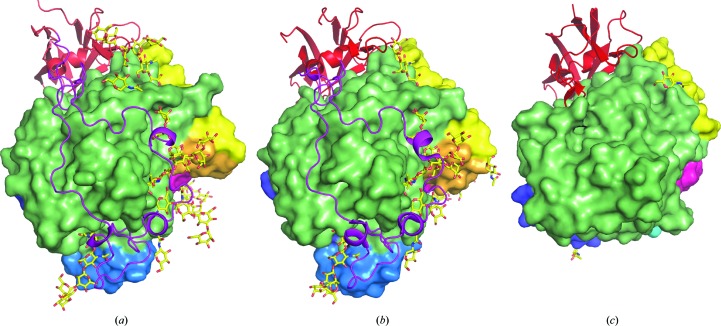
Domain 3 and loop V displayed in ribbon representation (red and magenta, respectively) for (*a*) *Nc*Cel3A, (*b*) *Re*Cel3A (PDB entry 5ju6; Gudmundsson *et al.*, 2016 ▸) and (*c*) *Hj*Cel3A (PDB entry 3zyz; Karkehabadi *et al.*, 2014 ▸). The N-glycans attached to the three structures are displayed as yellow sticks and other domains are represented as surfaces.

Domain 3 is an FnIII-like or immunoglobulin s-type domain (residues 650–857) and was first observed in GH3 in the *Tn*Bgl3B structure (Pozzo *et al.*, 2010 ▸). The FnIII domain is a β-sandwich composed of two layers of β-sheets of three and four β-strands, respectively (Fig. 5 ▸). The extended loop V, which was first observed in the structures of *Aa*BGLI and *Re*Cel3A, encompasses domain 1 and interacts with loop II on the opposite side of the molecule from domain 3. Loop V folds over three large N-glycan chains (bound to Asn61, Asn311 and Asn318). Several conserved aromatic residues π-stack with GlcNAc residues in loop V (Tyr706, Tyr708, Tyr723 and Phe730). Lima and coworkers proposed that the homologous loop V from *Aspergillus niger* Bgl1 (*An*Bgl1) is flexible and allows the FNIII domain to extend and bind to lignin, thus explaining the tadpole-like structure that was observed in SAXS experiments carried out with *An*Bgl1 (Lima *et al.*, 2013 ▸). We argue, however, that the domain reorganization speculated upon by Lima and coworkers is unlikely. Firstly, many of the conserved residues form seemingly crucial stacking inter­actions with N-glycans, a fact that is not accounted for in their model. Secondly, the presence of flexibility within a protein crystal usually results in poor or even no electron density. This is observed for instance in the *Ps*ExoP structure, the only published GH3 structure in which electron density is completely missing for one highly flexible domain even though it was expressed as part of the protein. We thus believe it is unlikely that loop V has this degree of flexibility in *Nc*Cel3A and other GH3 proteins that contain this loop.

### 3.7. Subsite −1 and catalytic residues   

The location of the catalytic centre subsite −1 of *Nc*Cel3A is positioned at the carboxyl side of domain 1. The two catalytic residues of *Nc*Cel3A were identified based on homology to other GH3 structures. The nucleophile is Asp276 and the acid/base is Glu505 (Fig. 3 ▸ *a*). No distinct density corresponding to a bound glucose was observed in subsite −1 of *Nc*Cel3A, in contrast to several other GH family 3 structures. Extra electron density is present in the −1 subsite that is insufficient for interpretation. This density may be owing to a partially bound buffer molecule, a polyethylene glycol (PEG) molecule and/or partial density of a glucose molecule.

### 3.8. Putative +1 and +2 subsites   

The putative +1 subsite of the *Nc*Cel3A structure is very similar to those of *Re*Cel3A and *Aa*BGL1, but differs from the suggested +1 subsite in *Hv*ExoI, which is lined by two tryptophan residues (Fig. 4 ▸ *c*). Hrmova and coworkers proposed this to be the basis of the broad substrate specificity of *Hv*ExoI (Hrmova *et al.*, 2002 ▸). Trp277 in *Nc*Cel3A corresponds to one of these tryptophan residues, but the side chain has shifted to become an essential part of the −1 subsite rather than the +1 subsite as in *Hv*ExoI. This shift causes a rearrangement of the core residues and contributes to the collapse of the TIM-barrel fold described above. In the collapsed TIM-barrel fold, a feature that seems to be shared by many fungal and bacterial β-glucosidases, the second barrel β-strand is shorter and antiparallel, which makes the −1 subsite wider compared with the active site in GH3 enzymes with a complete TIM-barrel fold. Similar to the structures of *Re*Cel3A and *Aa*BGL1, one side of the +1 subsite is formed by Phe301, which stacks with the side chain of Trp64, which is only slightly further away from the active site. Phe507 is situated on the opposite side of the +1 subsite and the active-site entrance. This phenylalanine seems to correspond to the ‘coin-slot’ Trp434 side chain in *Hv*Exo1. The corresponding residues in *Re*Cel3A and *Aa*BGL1 have almost the same side-chain conformations but are tyrosines (Tyr507 and Tyr511, respectively) in both these enzymes. In both the *Re*Cel3A and the *Aa*BGL1 structures the hydroxyl group of the tyrosine makes hydrogen-bond interactions with Asp433 and Asp437 in a potential +2 subsite (Fig. 4 ▸). In the *Nc*Cel3A structure the corresponding residue (Asp432) interacts with the side chain of Arg434, which should stabilize the aspartate residue and compensate for the slight increase of hydrophobicity in the +1 binding site. This arginine is not present in the *Re*Cel3A and *Aa*Bgl1 enzymes. It thus seems as if not only the presence of the aspartate residue but also its flexibility/stability may be important for substrate and/or product interaction in this class of β-glucosidases. Arg196 and Gln197 are two conserved residues in *Re*Cel3A and *Aa*BGL1 that make potentially important interactions with the substrate, forming part of the +1 subsite.

Previously, we have shown that *Re*Cel3A prefers cellobiose to cellotriose, while *Hj*Cel3A prefers the hydrolysis of slightly longer cello-oligosaccharides such as cellotriose and cello­tetraose (Karkehabadi *et al.*, 2014 ▸; Gudmundsson *et al.*, 2016 ▸). In analogy with the *Re*Cel3A structure, the plane of the Trp64 side chain in the *Nc*Cel3A structure has rotated almost 90° in the structure when compared with the corresponding tryptophan residue in the *Hj*Cel3A structure, and stacks with the side chain of Phe301. This puts the phenylalanine residue in subsite +1 rather than in a tentative +2 subsite, as in the structure of *Hj*Cel3A. Thus, similar to *Re*Cel3A the existence of a +2 subsite is less pronounced in *Nc*Cel3A than in *Hj*Cel3A and the enzyme may also have a substrate specificity similar to that of *Re*Cel3A.

## 4. Conclusions   

We have determined the structure of a glycoside hydrolase family 3 β-glucosidase, Cel3A from *N. crassa*, at 2.2 Å resolution and show that this β-glucosidase is structurally similar to two other fungal β-glucosidases: *A. aculeatus* BglI and *R. emersonii* Cel3A. These structures share several features that may be unique to this class of GH3 β-glucosidases. Most pronounced among these features are the likely dimeric form of the active enzyme and the large and seemingly conserved glycosylations. The structural analysis further showed that *Nc*Cel3A should have a similar substrate specificity to the previously structurally and biochemically characterized *Re*Cel3A.

## Supplementary Material

PDB reference: *Neurospora crassa* Cel3A, 5nbs

## Figures and Tables

**Table 1:** Data collection and processing Values in parentheses are for the outer shell.

Data collection	
Diffraction source	I911-3, MAX-lab
Wavelength (Å)	1.0
Temperature (K)	100
Detector	MAR Mosaic 225
Crystal-to-detector distance (mm)	198.59
Rotation range per image (°)	0.25
Total rotation range (°)	97.5
Space group	*P*2_1_2_1_2
*a*, *b*, *c* (Å)	142.9, 286.8, 58.0
α, β, γ (°)	90, 90, 90
Resolution range (Å)	47.00–2.25
Total No. of reflections	217078
No. of unique reflections	113592
Completeness (%)	99.2
Multiplicity	1.9 (1.9)
〈*I*/σ(*I*)〉	22.9 (4.3)
*R*_merge_ †	0.028 (0.38)
*R*_p.i.m._ ‡	0.028 (0.38)
*R*_r.i.m._ §	0.040 (0.53)
CC_1/2_ ¶	0.998 (0.825)
Overall *B* factor from Wilson plot (Å^2^)	29.7
Structure refinement	
Resolution range (Å)	286.8–2.25
Completeness (%)	99.2
No. of reflections, working set	107929 (7878)
No. of reflections, test set	5627 (417)
Final *R*_cryst_ ††	0.18 (0.30)
Final *R*_free_ ††	0.22 (0.35)
No. of non-H atoms	
Total	15120
Protein	13147
Carbohydrate	1021
Water	954
Model quality	
R.m.s. deviations	
Bonds (Å)	0.012
Angles (°)	1.625
Ramachandran plot‡‡	
Most favoured (%)	97
Allowed (%)	2.9
Pyranose conformations (total/percentage)§§	
Lowest energy conformation	83/100
Higher energy conformations	0.0/0

† *R*_merge_ = 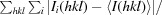 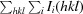, where *I*_*i*_(*hkl*) is the intensity of the *i*th measurement of an equivalent reflection with indices *hkl* and 〈*I*(*hkl*)〉 is the mean intensity of *I*_*i*_(*hkl*) for all *i* measurements.

‡ *R*_p.i.m._ is the precision-indicating (multiplicity-weighted) *R*_r.i.m._ (Diederichs & Karplus, 1997 ▸; Weiss, 2001 ▸).

§ *R*_r.i.m._ is the redundancy-independent (multiplicity-weighted) *R*_merge_ (Evans, 2006 ▸, 2012 ▸).

¶ CC_1/2_ is the correlation coefficient of the mean intensities between two random half-sets of data (Karplus & Diederichs, 2012 ▸; Evans, 2012 ▸).

†† *R*_cryst_ = 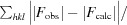 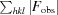; *R*_free_ is calculated in an identical manner using a randomly selected 5% of the reflections which were not included in the refinement.

‡‡ Calculated using a strict-boundary Ramachandran definition given by Kleywegt & Jones (1996 ▸).

§§ Calculated using the *Privateer* software (Agirre *et al.*, 2015 ▸) within *CCP*4*i*2 (Potterton *et al.*, 2018 ▸).
